# Supplementary material for: Rifampicin can induce antibiotic tolerance in mycobacteria via paradoxical changes in rpoB transcription
Source: Nat Commun. 2018 Oct 11;9:4218. doi: 10.1038/s41467-018-06667-3 (PMC6181997; doi:10.1038/s41467-018-06667-3)
Supplement: Supplementary file 1 — Supplementary Information [file 41467_2018_6667_MOESM1_ESM.pdf]

Rifampicin can induce antibiotic tolerance in mycobacteria via paradoxical changes in *rpoB* transcription

Zhu *et al*

Supplementary Information

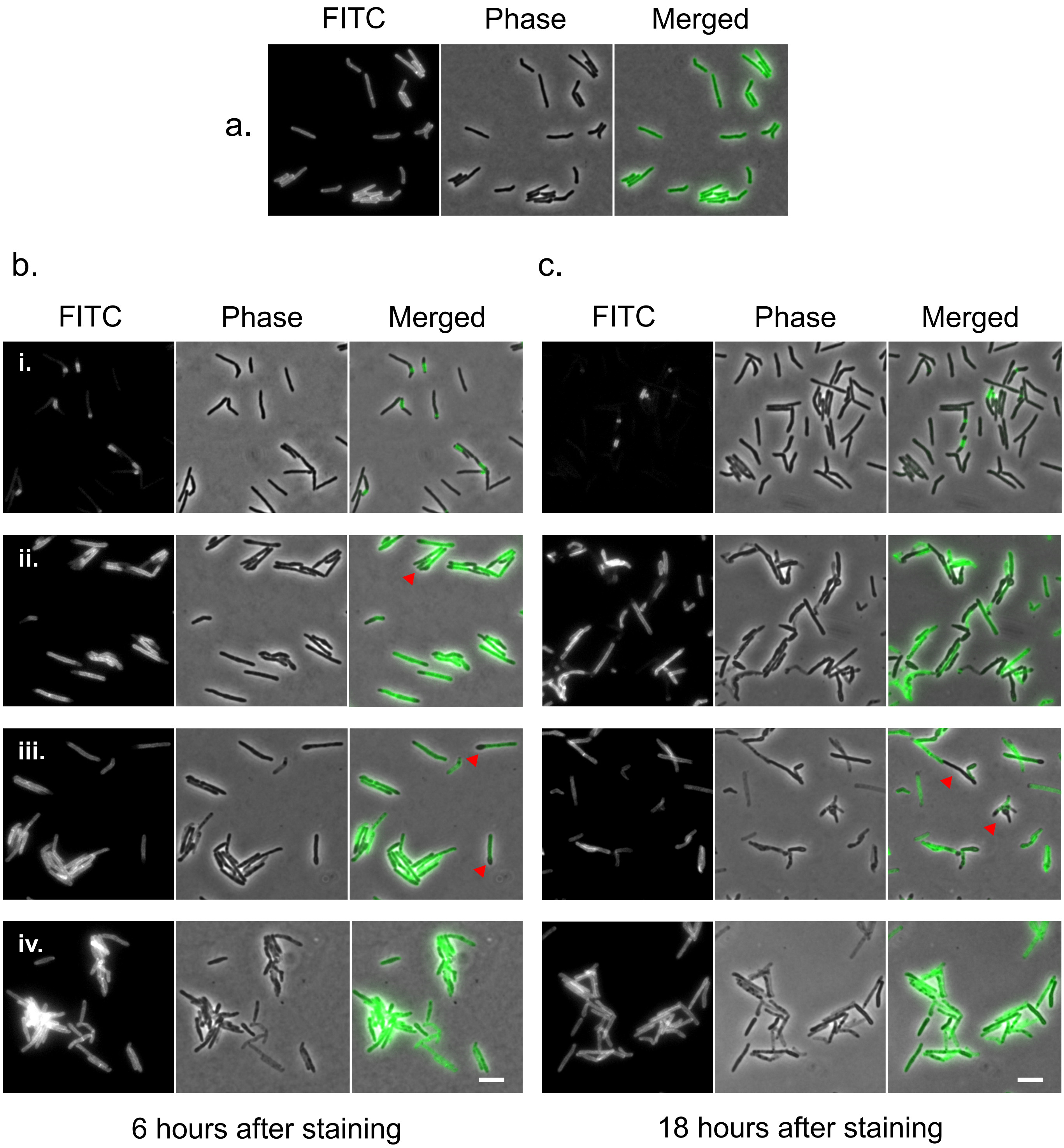

Supplementary Figure 1. Mycobacteria lose AF488 staining fluorescence upon polar growth. (a) Microscopy of cells immediately after staining. Fluorescence microscopy illustrating non-fluorescence of new growth at the poles (arrowheads) of some *M. smegmatis* cells following 6 hours (b) and 18 hours (c) exposure to 10µg/ml rifampicin. Scale bar, 5 µm.

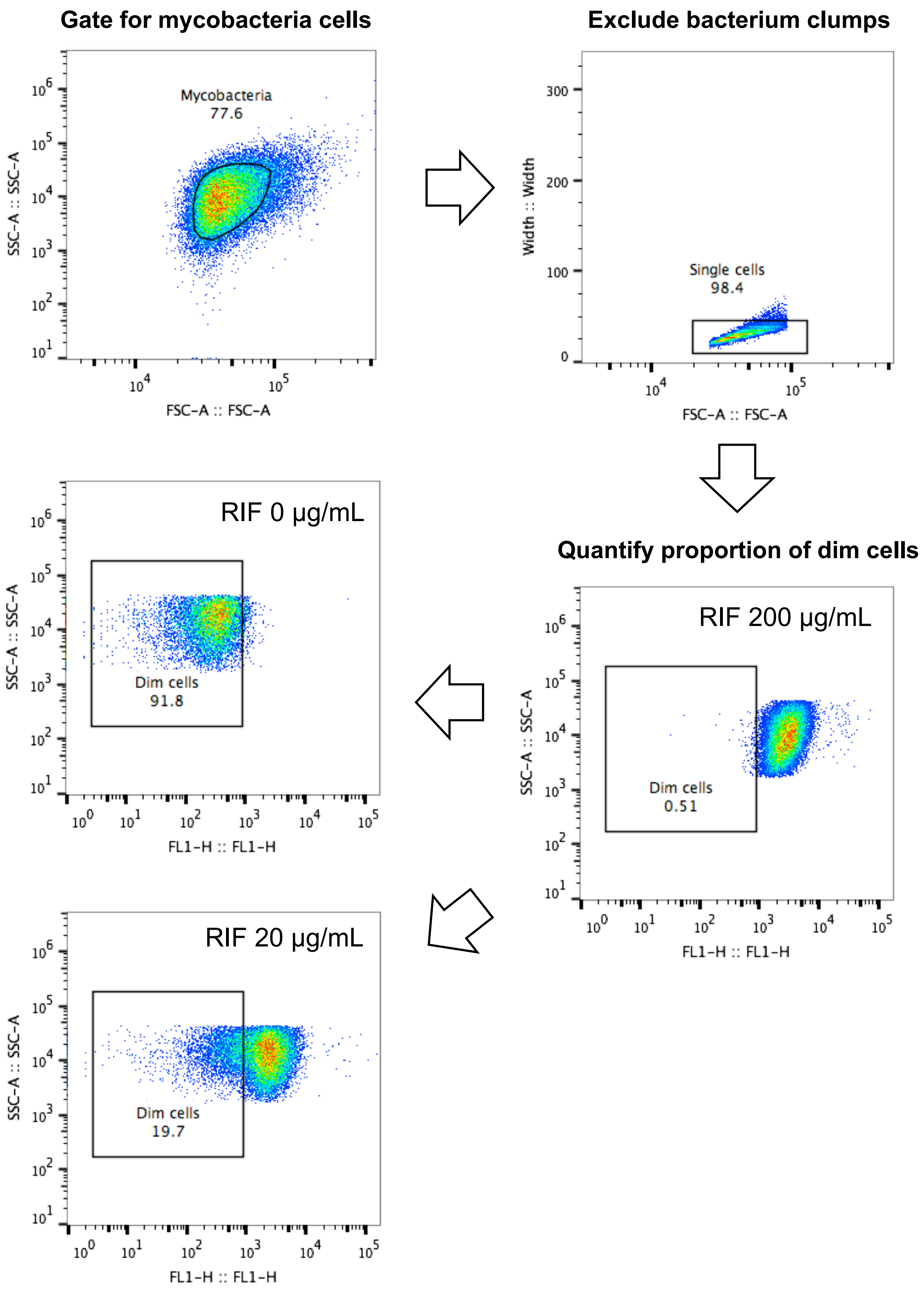

Supplementary Figure 2. Flow cytometry strategy for the fluorescence-dilution assay. Gating and fluorescence intensity strategy for scoring dim cells by flow cytometry following AF488 staining.

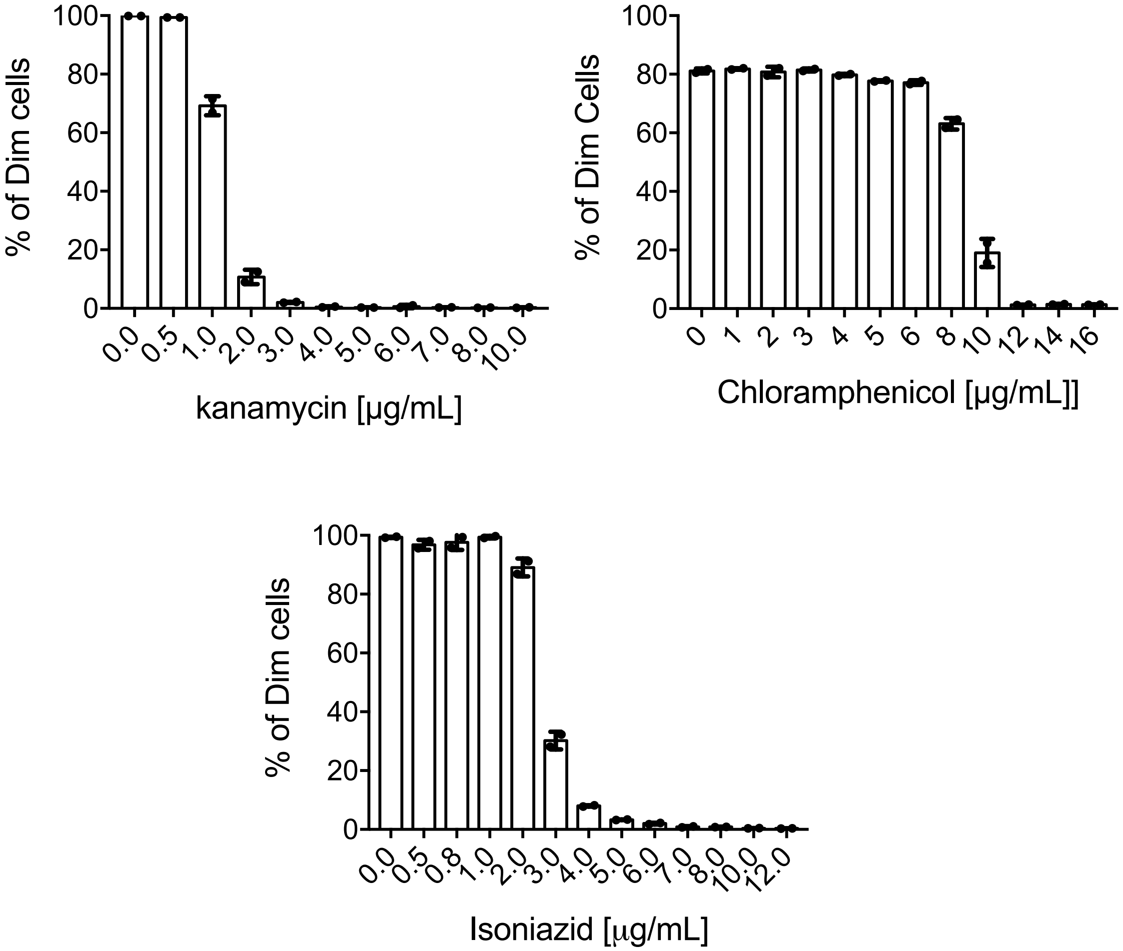

Supplementary Figure 3. Fluorescence-dilution assay for *M. smegmatis* exposed to chloramphenicol, kanamycin and isoniazid. Cells grown for 16 hours following staining with AF488 in 7H9 and indicated concentration of antibiotics prior to flow cytometric analysis.

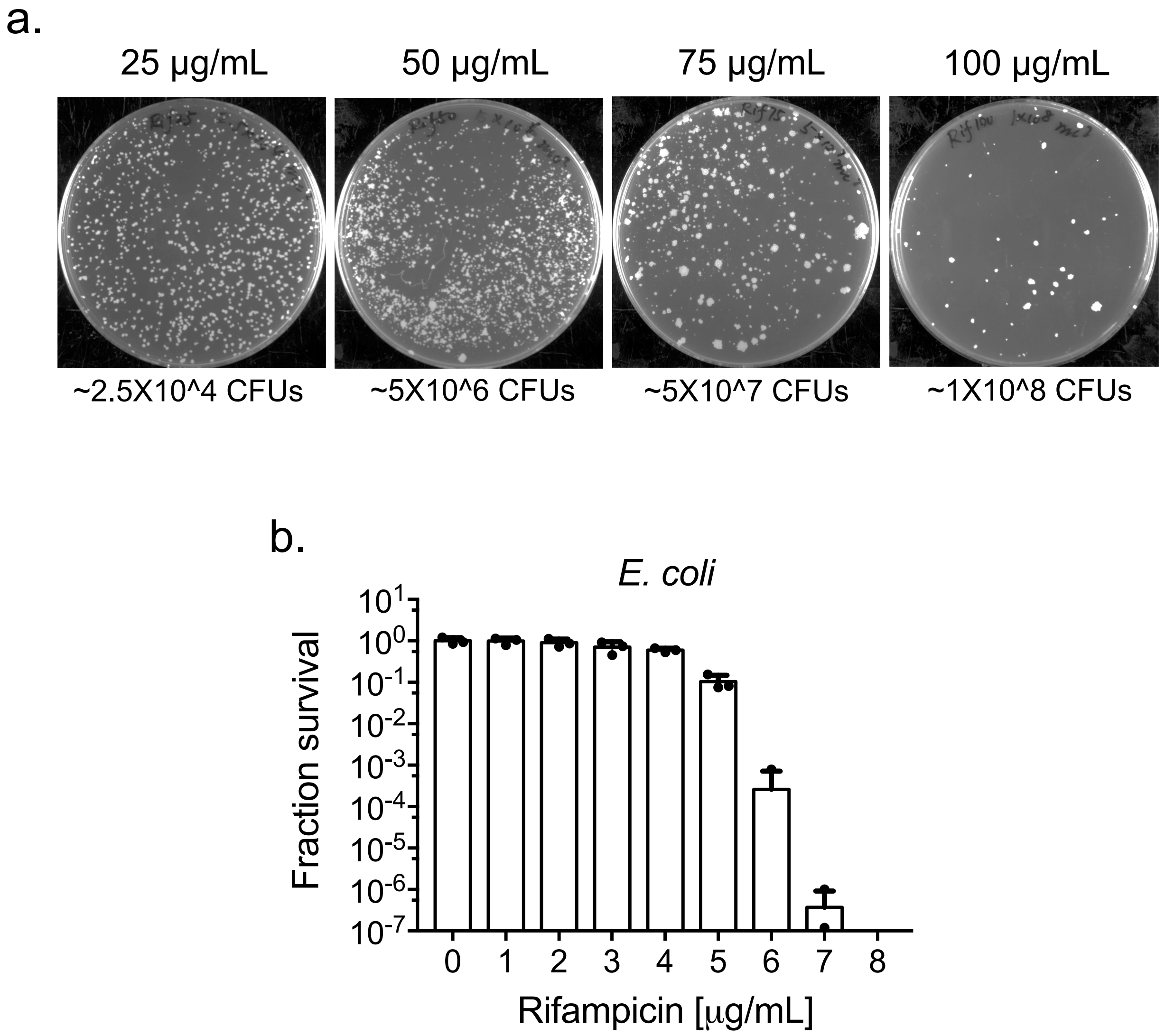

Supplementary Figure 4. Phenotypically resistant mycobacteria can form colonies on rifampicin-agar medium (a) *M. smegmatis* at the indicated colony forming units was plated on rifampicin-agar at varying concentrations to identify growers. (b) *E. coli* were plated on rifampicin-agar at varying concentrations and the fractional survival (number of colonies on rifampicin-agar compared with non-selective medium) calculated. Result represents 3 biological triplicates per concentration.

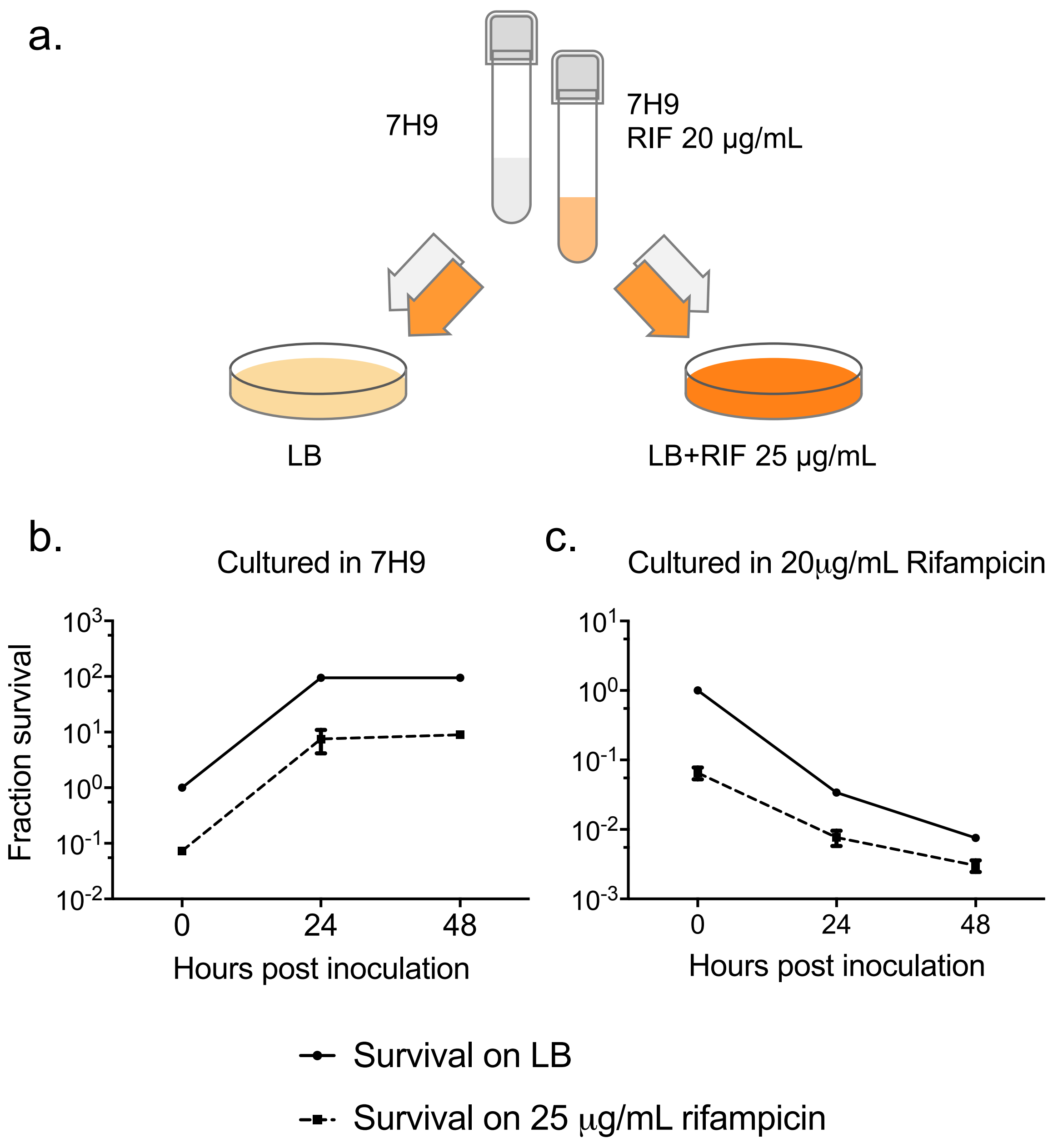

Supplementary Figure 5. Mycobacterial adaptive phenotypic resistance to rifampicin. (a) Schematic illustrating experiment. *M. smegmatis* was cultured in complete 7H9 medium +/- rifampicin and then aliquots of each culture were plated on both non-selective (LB) agar or rifampicin-agar (LB+RIF) to calculate fractional survival. Relative CFUs compared with time = 0 (time of inoculation into fresh medium +/- antibiotic), plated on non-selective agar of aliquots grown in 7H9 without rifampicin prior to plating (b) or grown in 7H9-rifampicin (c) prior to plating. Note the fractional survival on rifampicin of cultures grown in antibiotic-free 7H9 is constant over time, but converges (indicating relatively increased survival and growth) when pre-cultured in rifampicin.

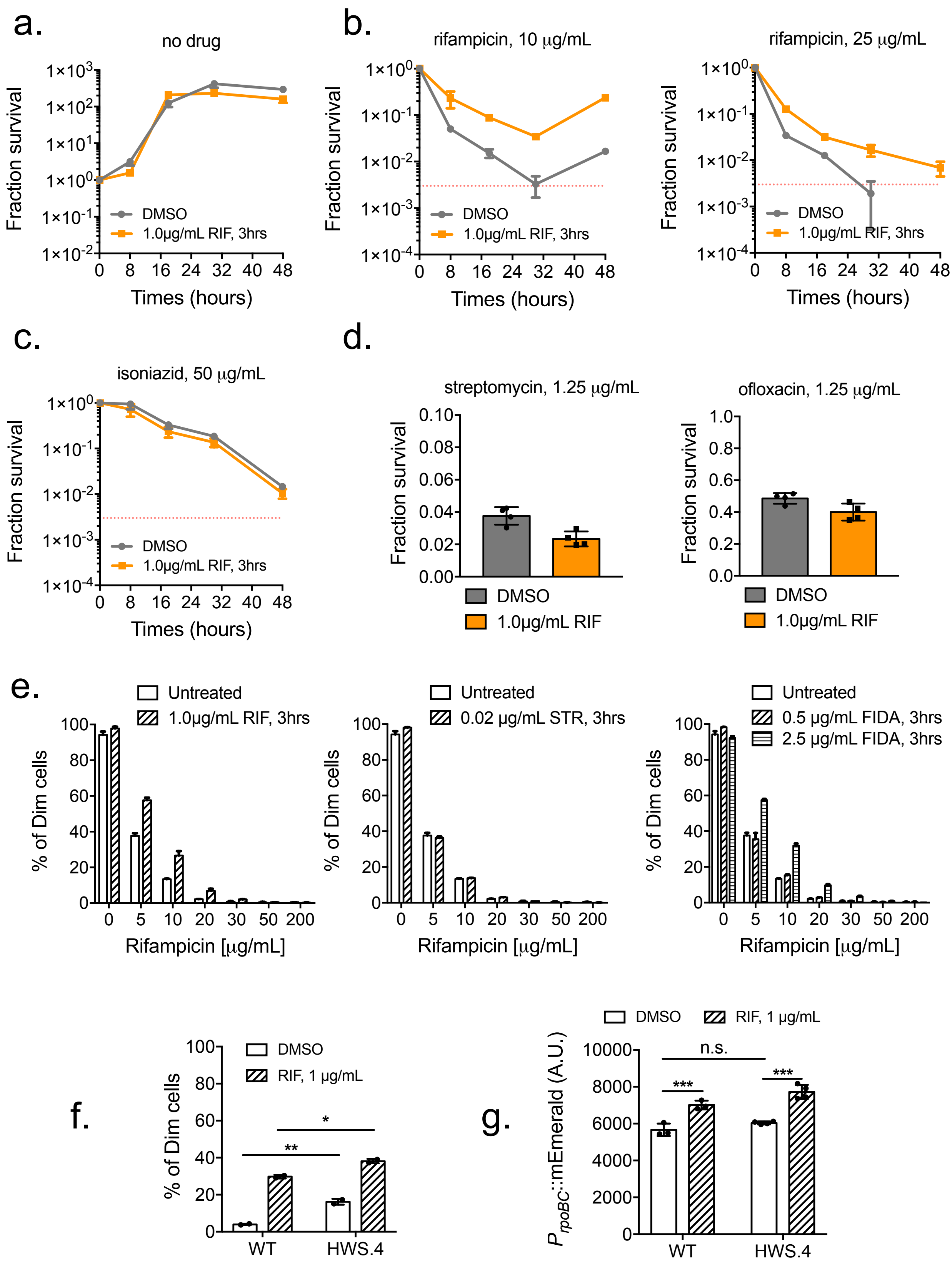

a.
no drug
Fraction survival
Times (hours)
DMSO
1.0μg/mL RIF, 3hrs
b.
rifampicin, 10 μg/mL
rifampicin, 25 μg/mL
c.
isoniazid, 50 μg/mL
d.
streptomycin, 1.25 μg/mL
ofloxacin, 1.25 μg/mL
1.0μg/mL RIF
e.
Untreated
1.0μg/mL RIF, 3hrs
0.02 μg/mL STR, 3hrs
0.5 μg/mL FIDA, 3hrs
2.5 μg/mL FIDA, 3hrs
% of Dim cells
Rifampicin [μg/mL]
f.
DMSO
RIF, 1 μg/mL
WT
HWS.4
g.
n.s.
P_{rpoBC}::mEmerald (A.U.)

Supplementary Figure 6. RNAP inhibiting drugs specifically trigger rifampicin tolerance. *M. smegmatis* was exposed to sub-MIC (1µg/ml) rifampicin or DMSO and then after washing grown in 7H9 alone (a) or in the presence of bulk-lethal concentrations of rifampicin (b) or isoniazid (c). Aliquots were taken at indicated time-points, washed and plated on non-selective medium. Relative survival data represent 4 biological replicates. (d) Relative survival at 8 hours post oflaxicin or streptomycin treatment for a similar experiment. (e) Fluorescence dilution assay for *M. smegmatis* pre-exposed to sub-MIC rifampicin, streptomycin and fidaxomicin and then subsequent culture in rifampicin at indicated concentrations for 16 hours prior to flow cytometry. (f) Fluorescence dilution assay and *rpoB* promoter expression (g) of an *M. smegmatis* strain with a *gatA*-V405D mutation conferring high specific mistranslation rates[14]. The results represent the biological duplicates (f) or triplicates (g) of each experiment. * $p<0.05$, ** $p< 0.01$ and *** $p< 0.001$ by Student's t-test.

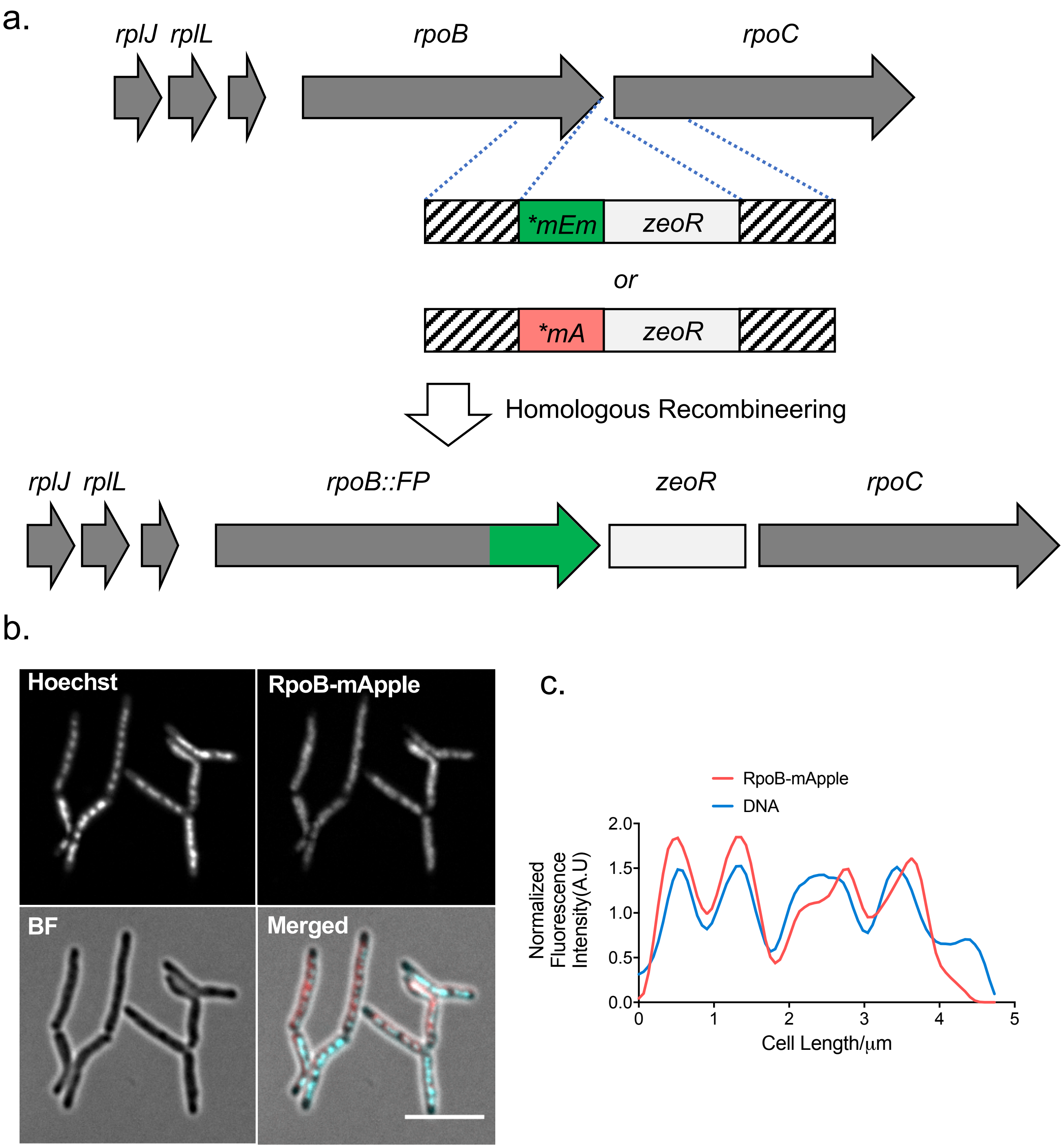

Supplementary Figure 7. Construction of *M. smegmatis rpoB-mFluorescent Protein* strains to follow RpoB accumulation. (a) Schematic showing the chromosomal location of the *rpoB-rpoC* operon and genetic strategy by homologous recombination to construct the fluorescent-protein (FP) tagged RpoB at its native location. mEm (monomeric Emerald fluorescent protein), mA (monomeric Apple fluorescent protein). zeoR: zeocin resistance cassette. (b) Fluorescence microscopy showing DNA staining by Hoescht 33342 (DAPI channel) and RpoB-mApple (TRITC channel), scale bar, 5 μm. (c) Relative co-localization of blue and red fluorescence (DNA and RpoB-mApple respectively) in one representative cell.

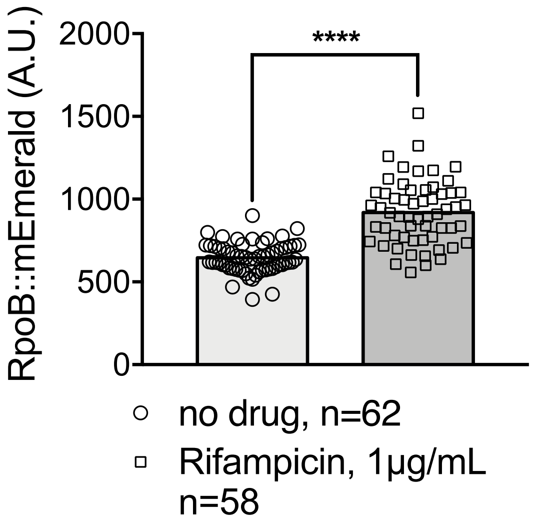

Supplementary Figure 8. SubMIC rifampicin exposure upregulates RpoB. Relative fluorescence intensity of three biological replicates treated as in Fig. 1k and fixed prior to microscopy. Each replicate represents cells in one high-powered field (individual points) and bar represents mean fluorescent intensity. **** $p < 0.0001$ by Mann-Whitney's U test.

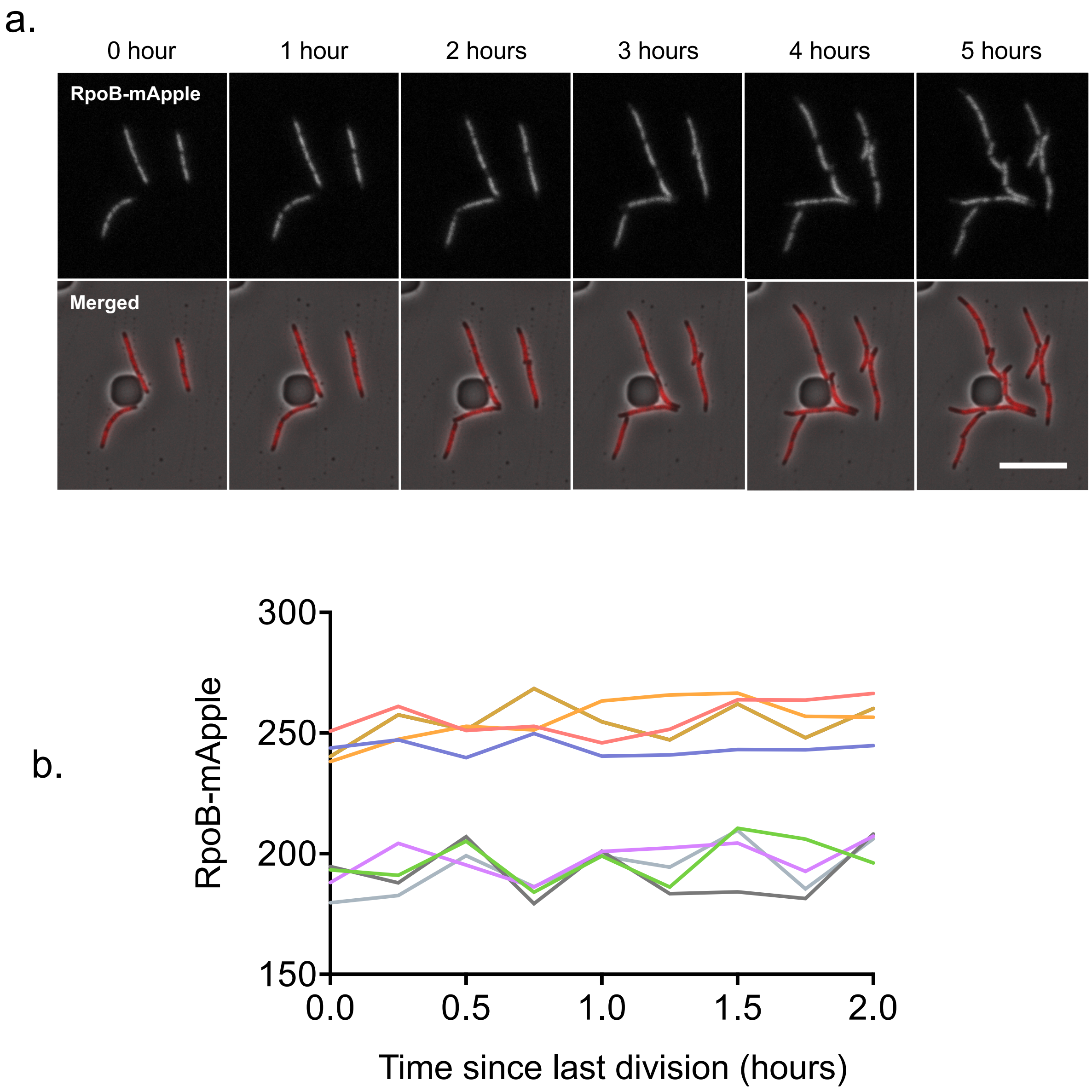

Supplementary Figure 9. RpoB abundance does not change significantly without rifampicin exposure. (a) Time-lapse fluorescence microscopy of a micro-colony in a flow chamber without rifampicin. (b) Mean fluorescent intensity (MFI) of RpoB-mApple in 9 representative *M. smegmatis* cells over time showing variable, but stable RpoB expression over 1 cell cycle. Scale bar, 10 μm.

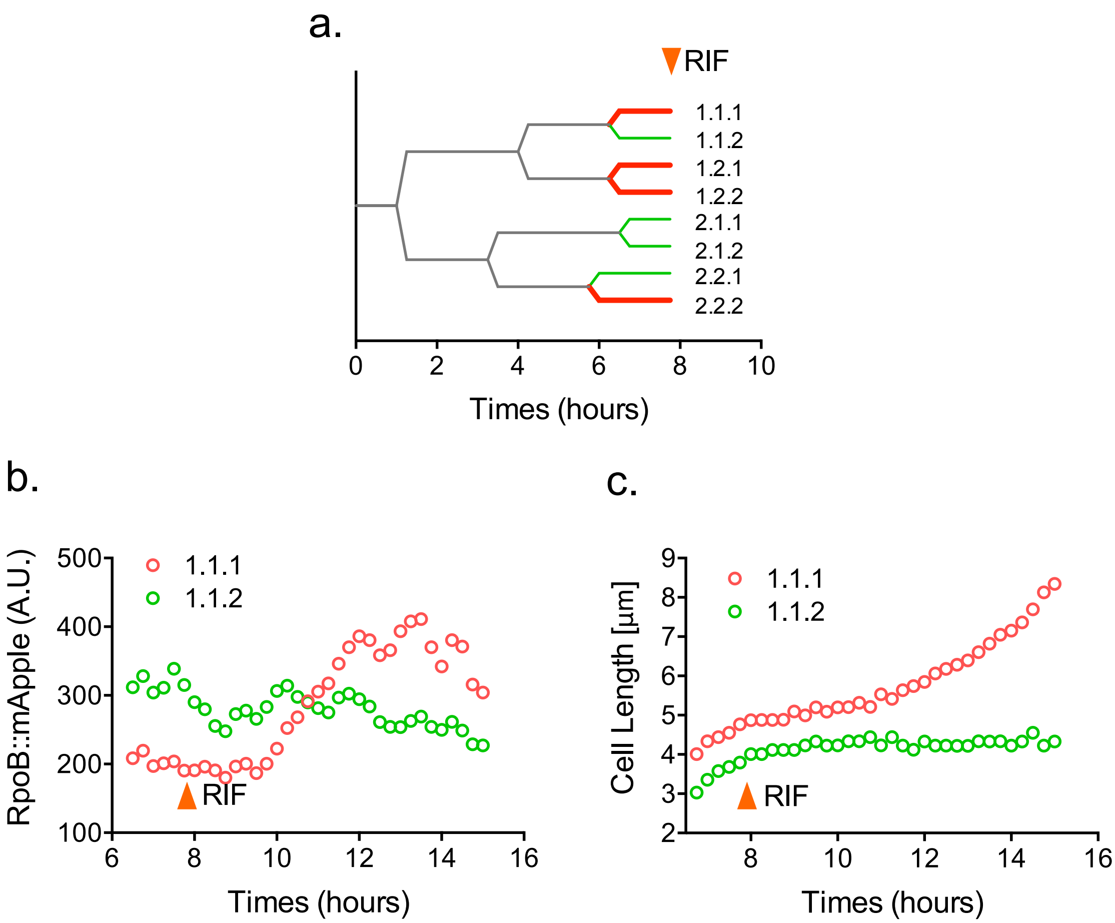

Supplementary Figure 10. Phenotypically resistant growers upregulate and accumulate RpoB. (a) Pedigree tree of the microcolony illustrated in Fig. 2c and Supplementary movie 1. Bifurcations represent cell division. Lineages are described at the right of the graph e.g. 1.2.1 represents daughter 1 from the first division, daughter 2 from the second division and daughter 1 from the third division. Cells with subsequent RpoB accumulation after rifampicin exposure (initiated at 8 hour time-point) are illustrated in red, the other cells in green. Red-fluorescence intensity (representing RpoB-mApple) (b) and cell length (c) of two siblings (the top branch lineage of (a)) with differing cell-fates following rifampicin exposure. Each circle represents a discrete measurement.

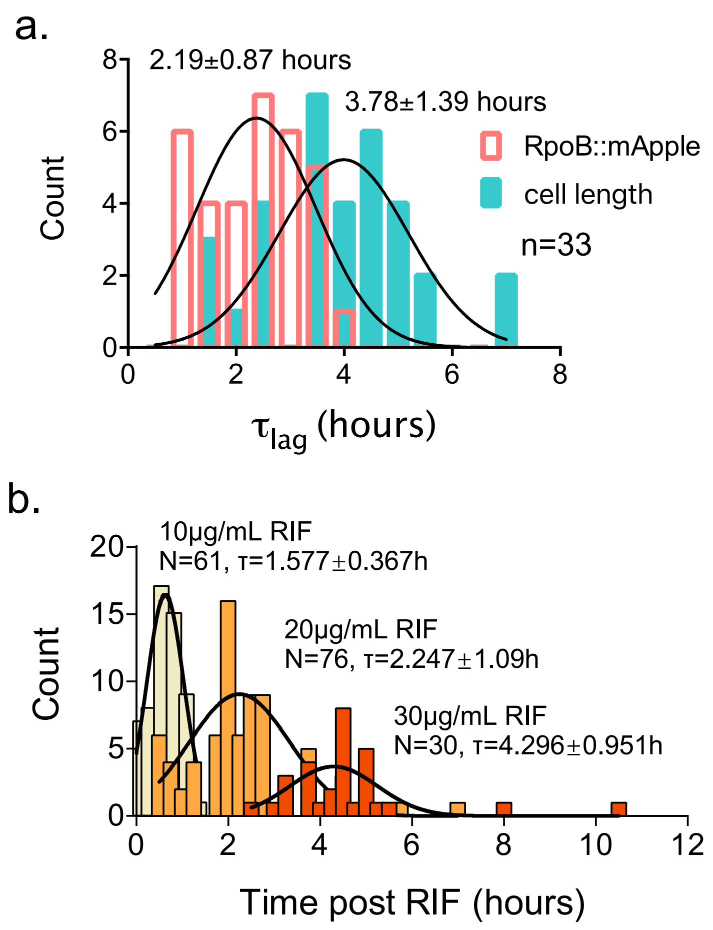

Supplementary Figure 11. RpoB accumulation and cell elongation occur after rifampicin exposure, after time-lag in proportion to the antibiotic concentration. (a) Histogram of lag time for RpoB accumulation – red bars, and increase in cell length – cyan bars (see Methods) following 20 μg/ ml rifampicin exposure. Trend lines are shown in black and fit a Gaussian distribution. (b) The lag time for RpoB accumulation is positively correlated with rifampicin concentration.

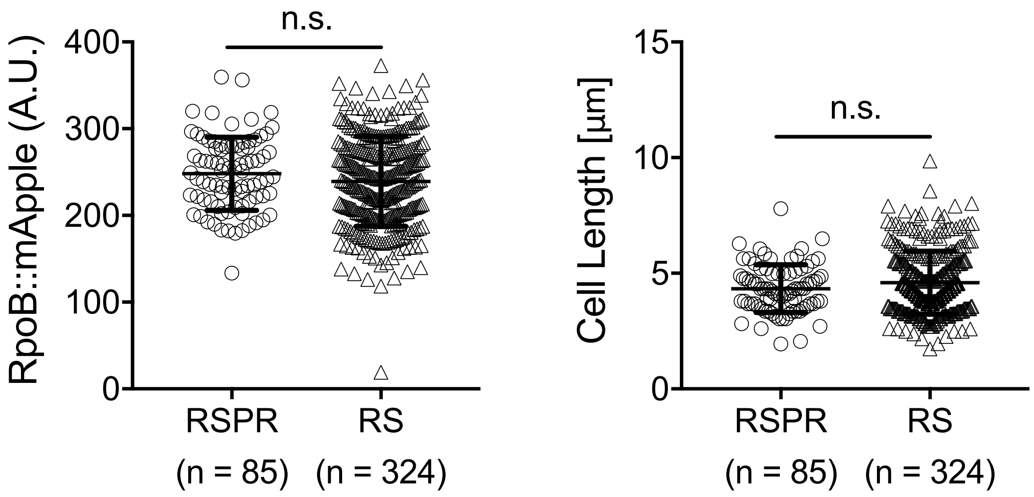

Supplementary Figure 12. RpoB abundance or cell size prior to rifampicin exposure do not predict RSPR. Relative fluorescence intensity (representing RpoB-mApple) (a) and cell size (b) of individual *M. smegmatis* cells at time immediately prior to addition of rifampicin (20µg/ ml) to the flow chamber. RSPR or not (rifampicin-susceptible – RS) is scored by growth and RpoB accumulation following rifampicin exposure. Mean +/- SD shown. n.s. = no significant difference by Kolmogorov-Smirnov test.

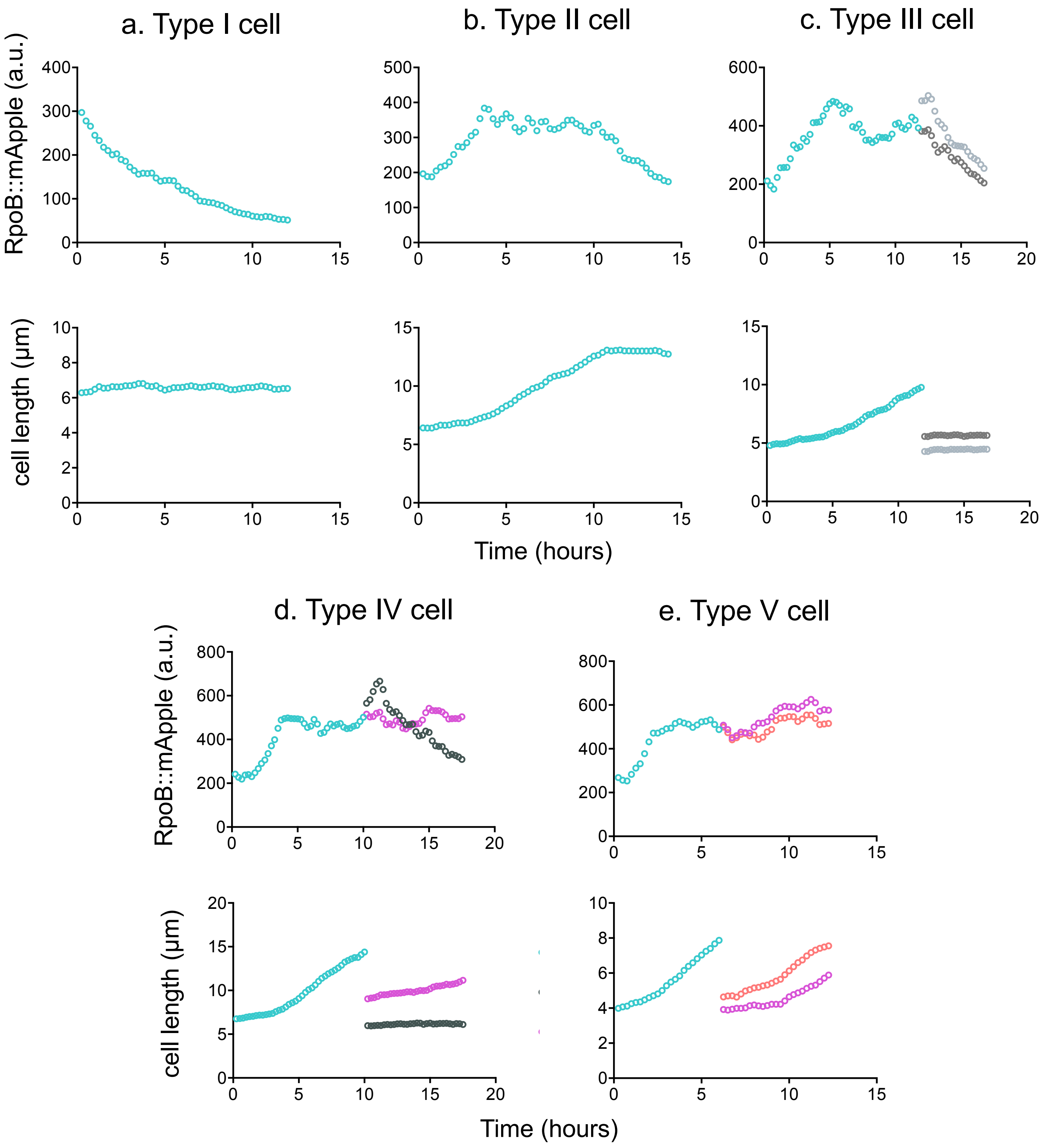

Supplementary Figure 13. Categorization of rifampicin-sensitive and RSPR responses following lethal rifampicin exposure. Relative cellular RpoB-mApple abundance and cell length of different types of response to 16 hours of 20 µg/ml rifampicin. Each panel (a-e) are data from one cell that represents the five differing responses of mother cells (blue circles) and progeny (red circles – RSPR daughters, grey circles – non-RSPR daughters). Circles represent a single measurement.

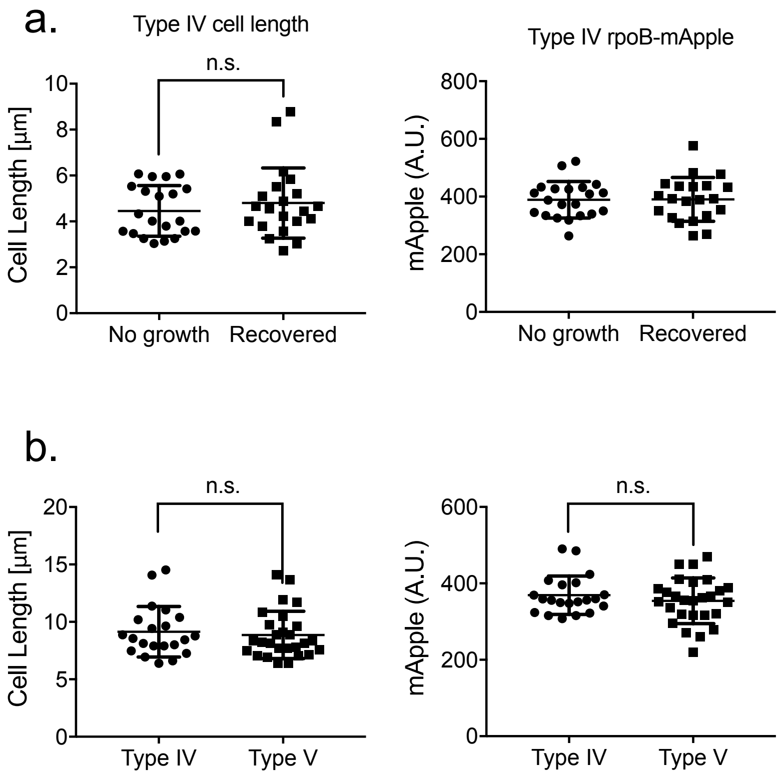

Supplementary Figure 14. Differences in cell-size and RpoB content cannot predict divergent cell-fate of sister mycobacteria in rifampicin. (a) Cell-size (left panel) and RpoB-mApple (right panel) were no different between daughters of Type IV cells with divergent fates in rifampicin (b) Similar measurements comparing Type IV and Type V cells immediately prior to rifampicin exposure could not predict the different outcomes in rifampicin. All these data refer to the experiment in Supplementary Fig. 13. ns = not significant by Mann-Whitney's U test.

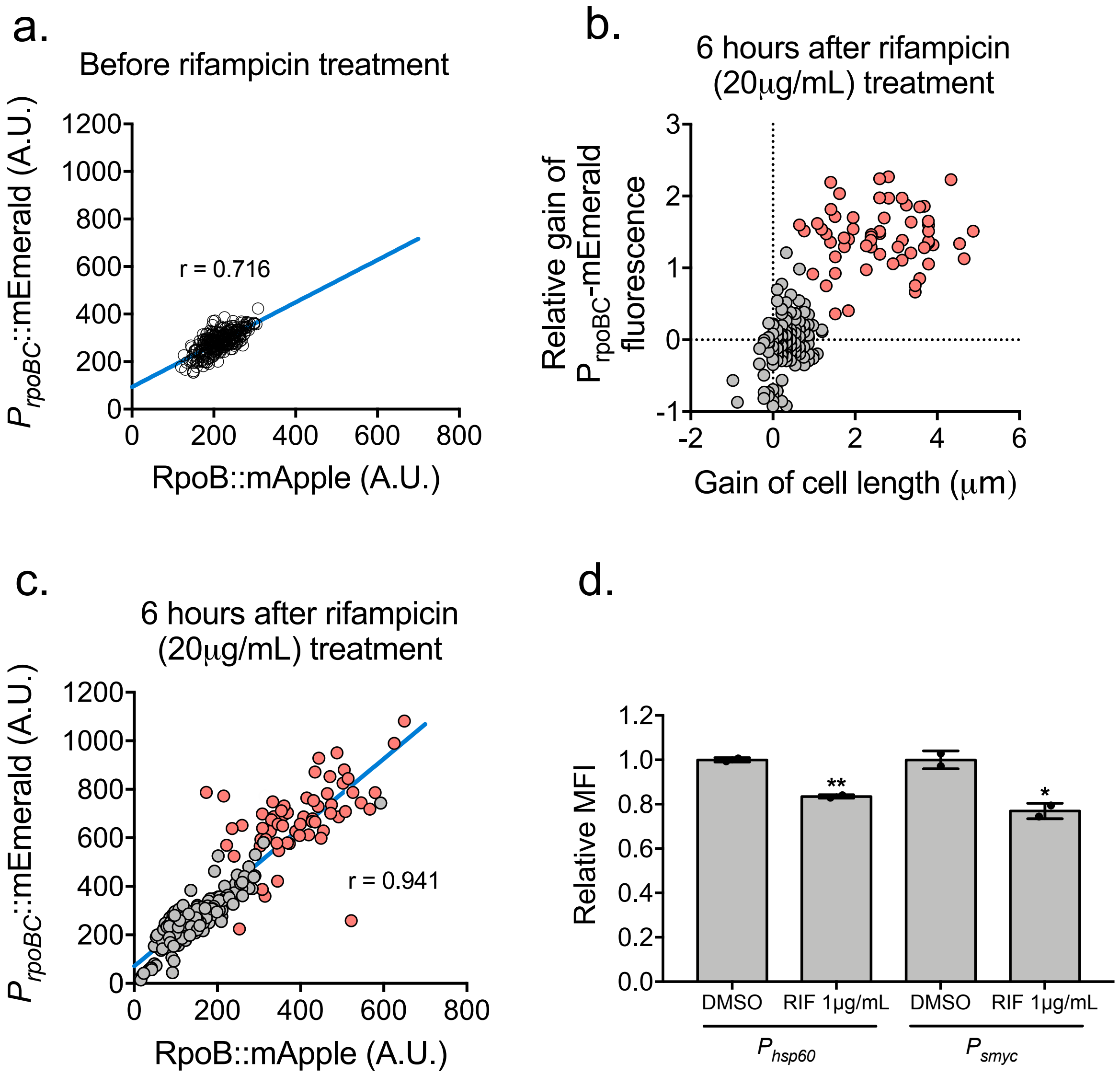

Supplementary Figure 15. Rifampicin exposure leads to divergent RpoB expression – increased expression in growers and decreased expression in rifampicin-susceptible cells. (a) Scatter plot of relative red (RpoB-mApple) and green (P_{rpoBC}*-driven* mEmerald) fluorescence before rifampicin treatment of 267 cells. Data are derived from experiment illustrated in Fig. 2c. (b) Scatter plot of growth versus green fluorescence of these cells following 6 hours of treatment with rifampicin. Since some cells divided, the number of cells following rifampicin treatment are greater than before rifampicin treatment. Cells that grew are filled in solid red. (c) Scatter plot of red versus green fluorescence as above, for the cells in (b) above. r represents Pearson correlation. (d) Mean fluorescence intensity (MFI) of P_{hsp60}- or P_{smyc}-driven mEmerald following 3 hours exposure to sub-MIC concentrations of rifampicin (RIF – 1μg/ml relative to vehicle as measured by flow cytometry. Bars represent biological duplicates. * $p<0.05$, ** $p< 0.01$ by Student's t-test.

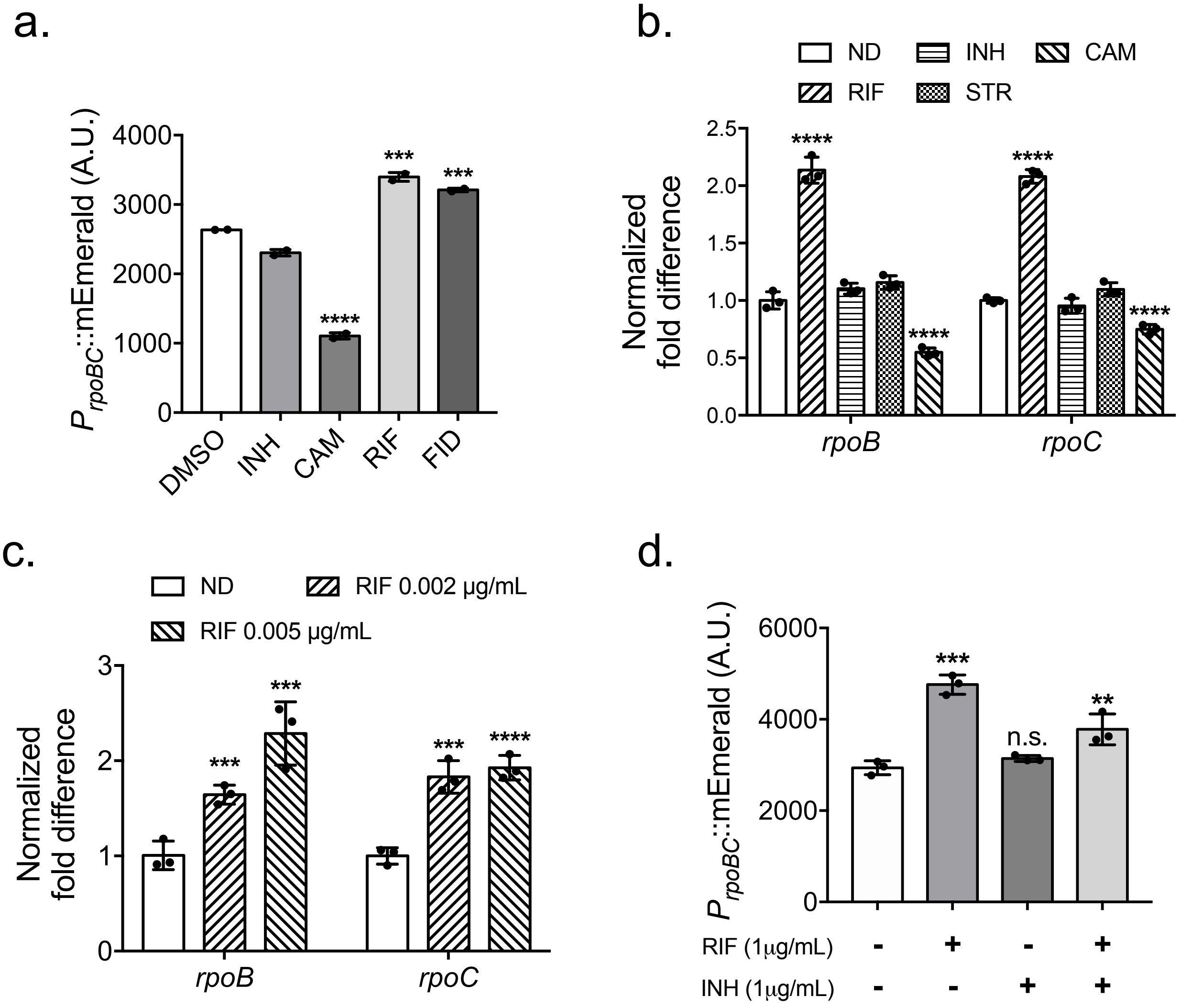

Supplementary Figure 16. RNAP targeting antibiotics specifically upregulate *rpoB* expression. (a) *M. smegmatis* expressing the P_{rpoBC}*-driven* mEmerald reporter was exposed to sub-MIC antibiotics as (a) as well as fidaxomicin (FID) at 2.5µg/ml and green fluorescence measured, representing *rpoB* promoter expression. Bars represent biological duplicates. (b) Quantitative PCR of mRNA transcripts of *rpoB* and *rpoC* relative to no drug (ND) control following 3 hours sub-MIC exposure of wild-type *M. smegmatis* to rifampicin (RIF, 1µg/ml), isoniazid (INH, 1µg/ml), chloramphenicol (CAM, 2.5µg/ml), streptomycin (STR, 0.02µg/ml). (c) Quantitative PCR of mRNA transcripts of *rpoB* and *rpoC* relative to no drug (ND) control following overnight sub-MIC exposure of wild-type BCG to rifampicin at indicated concentrations. Bars represent biological triplicates. (d) Co-exposure of *M. smegmatis* as in (a) to sub-MIC rifampicin and isoniazid together does not abolish upregulation of *rpoB* promoter activity. Bars represent biological triplicates. n.s. = not significant by Student's t-test. * p<0.05, ** p< 0.01 and *** p< 0.001 by Student's t-test.

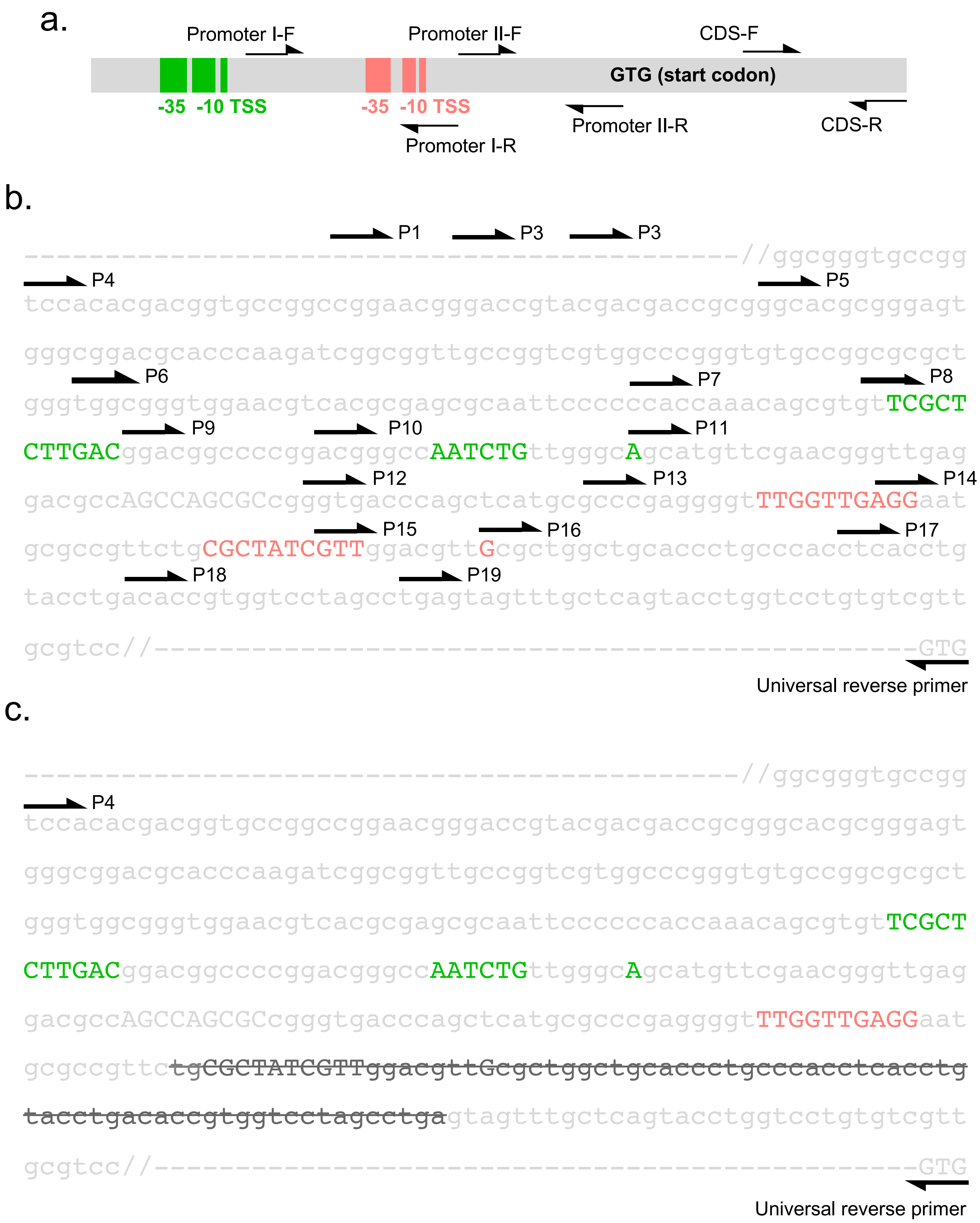
a.
Promoter I-F
Promoter II-F
CDS-F
GTG (start codon)
-35 -10 TSS
-35 -10 TSS
Promoter II-R
CDS-R
Promoter I-R
b.
P1
P3
P3
--//ggcgggtgccgg
P4
P5
tccacacgacggtgccggccggaacgggaccgtacgacgaccgcgggcacgcgggagt
gggcggacgcacccaagatcggcggttgccggtcgtggcccgggtgtgccggcgcgct
P6
P7
P8
gggtggcgggtggaacgtcacgcgagcgcaattccccccaccaaacagcgtgtTCGCT
P9
P10
P11
CTTGACggacggccccggacgggccAATCTGttgggcAgcatgttcgaacgggttgag
P12
P13
P14
gacgccAGCCAGCGCcgggtgacccagctcatgcgcccgaggggtTTGGTTGAGGaat
P15
P16
P17
gcgccgttctgCGCTATCGTTggacgttGcgctggctgcaccctgcccacctcacctg
P18
P19
tacctgacaccgtggtcctagcctgagtagtttgctcagtacctggtcctgtgtcgtt
gcgtcc//--GTG
Universal reverse primer
c.
--//ggcgggtgccgg
P4
tccacacgacggtgccggccggaacgggaccgtacgacgaccgcgggcacgcgggagt
gggcggacgcacccaagatcggcggttgccggtcgtggcccgggtgtgccggcgcgct
gggtggcgggtggaacgtcacgcgagcgcaattccccccaccaaacagcgtgtTCGCT
CTTGACggacggccccggacgggccAATCTGttgggcAgcatgttcgaacgggttgag
gacgccAGCCAGCGCcgggtgacccagctcatgcgcccgaggggtTTGGTTGAGGaat
gcgccgttctgCGCTATCGTTggacgttGcgctggctgcaccctgcccacctcacctg
tacctgacaccgtggtcctagcctgagtagtttgctcagtacctggtcctgtgtcgtt
gcgtcc//--GTG
Universal reverse primer

Supplementary Figure 17. Mapping of the two mycobacterial *rpoB-rpoC* promoters. (a) Schematic of the primers used for quantitative reverse-transcribed PCR to measure relative expression of transcripts from the promoter region of *rpoB-rpoC* for experiment in Fig. 3c. (b) Schematic of the location of primers to make the truncated promoter constructs to map the mycobacterial *rpoB-rpoC* promoters for Fig. 3d. The -35 and -10 elements and transcription start sites (TSS) of the two promoters are highlighted in red (Promoter I) and blue (Promoter II). (c) Schematic illustrating strategy to make the Promoter I only constructs (Supplementary Fig. 19a). The deleted DNA region is shown by struck-through letters.

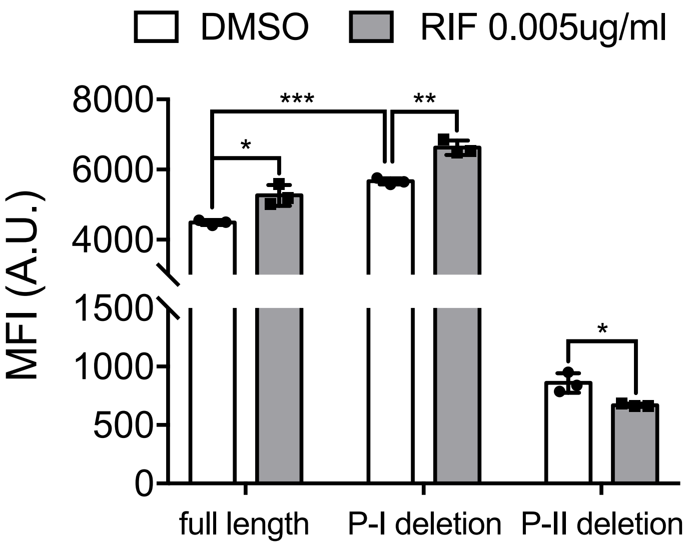

Supplementary Figure 18. Truncated promoter-driven expression of *rpoB* in BCG. Full-length, promoter I deletion and promoter I and II deletion constructs of P_{rpoBC}*-driven mEmerald* for the *M. tuberculosis/* BCG *rpoB-rpoC* promoter were constructed and transformed into BCG. Promoter activity +/- subMIC rifampicin overnight were measured using the green channel by flow cytometry. Bars represent biological triplicate. * p<0.05, ** p< 0.01, *** p<0.001 by Student's t-test.

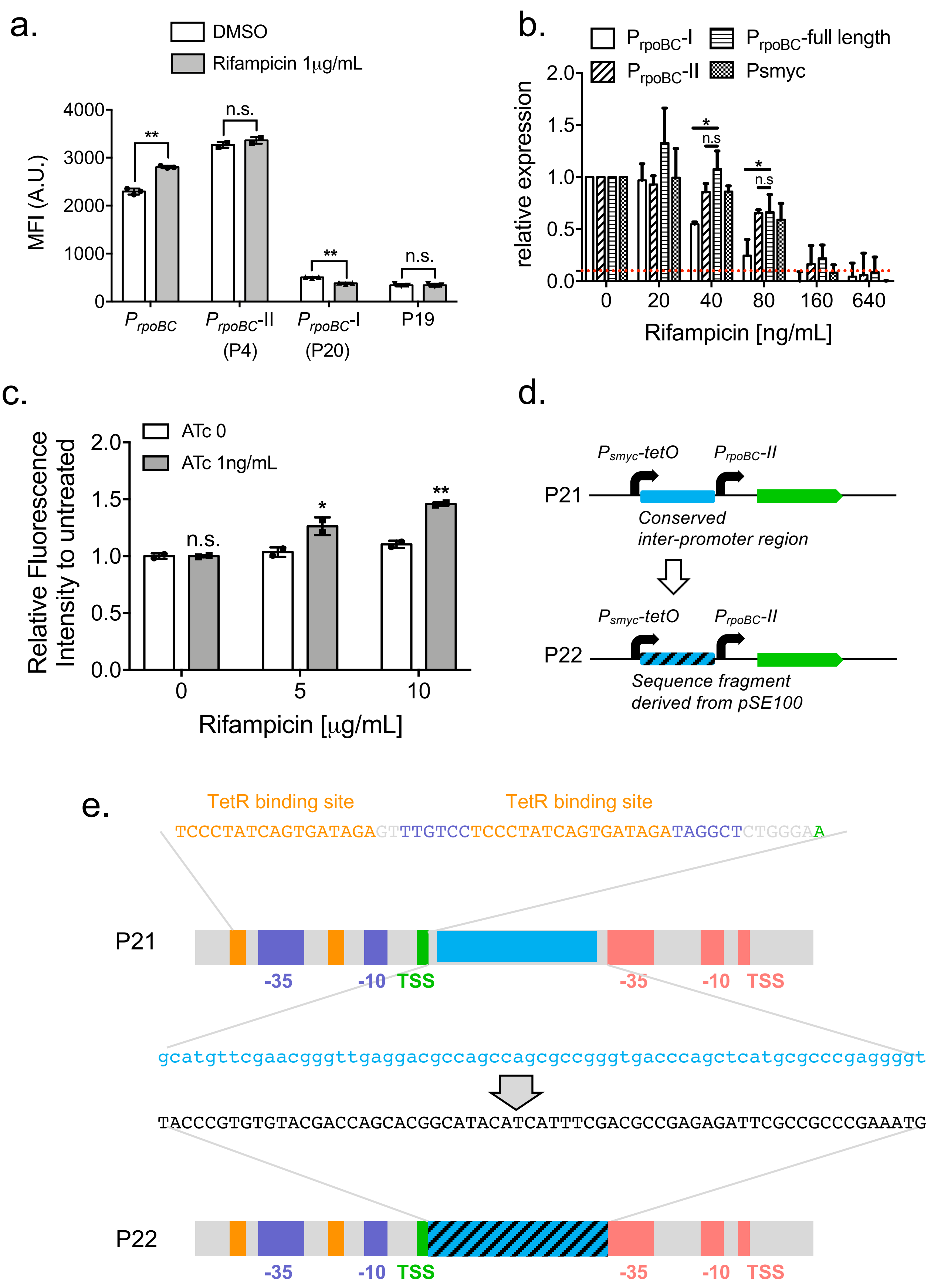
a.
DMSO
Rifampicin 1μg/mL
MFI (A.U.)
4000
3000
2000
1000
0
**
n.s.
**
n.s.
PrpoBC
PrpoBC-II
(P4)
PrpoBC-I
(P20)
P19
b.
PrpoBC-I
PrpoBC-II
PrpoBC-full length
Psmyc
relative expression
2.0
1.5
1.0
0.5
0.0
*
n.s
*
n.s
0
20
40
80
160
640
Rifampicin [ng/mL]
c.
ATc 0
ATc 1ng/mL
Relative Fluorescence Intensity to untreated
2.0
1.5
1.0
0.5
0.0
n.s.
*
**
0
5
10
Rifampicin [μg/mL]
d.
Psmyc-tetO
PrpoBC-II
P21
Conserved inter-promoter region
Psmyc-tetO
PrpoBC-II
P22
Sequence fragment derived from pSE100
e.
TetR binding site
TetR binding site
TCCCTATCAGTGATAGAGTTTGTCCTCCCTATCAGTGATAGATAGGCTCTGGGAA
P21
-35
-10
TSS
-35
-10
TSS
gcatgttcgaacgggttgaggacgccagccagcgccgggtgacccagctcatgcgcccgaggggt
TACCCGTGTGTACGACCAGCACGGCATACATCATTTCGACGCCGAGAGATTCGCCGCCCGAAATG
P22
-35
-10
TSS
-35
-10
TSS

Supplementary Figure 19. Mapping of the two mycobacterial *rpoB-rpoC* promoters. (a) Relative fluorescence of mEmerald measured by flow cytometry driven by the full-length *rpoB-rpoC* promoter, missing just Promoter I (P4), missing just Promoter II (P20) both promoters missing – see Fig. 3d, and a Promoter I only construct (see Supplementary Fig. 17c) +/- 1µg/ml rifampicin. Bars represent 3 replicates. ** p< 0.01, n.s. no significant difference by Student's t-test. (b) *In-vitro* transcription assay of dsDNA templates of the Msm Promoter I, Promoter II or full *rpoB-rpoC* promoter (Promoter I+II) sequences and a control *Psmyc* promoter +/- rifampicin at indicated concentrations (see Methods). Each bar represents 3 replicates. Dashed red line represents limit of detection by the assay. * p<0.05 by Student's t-test. (c) The relative MFI of RpoB^{L511P}-mEmerald, P_{tet}(*rpoB*^{WT}) in response to rifampicin or vehicle and +/- ATc at indicated concentrations. Bars represent duplicate measurements. ** p< 0.01, n.s. no significant difference by Student's t-test. (d) Schematic outlining the construction of the chimeric promoter constructs used in Fig. 3f. The Psmyc promoter is flanked by *tetR* binding sequences, allowing tetracycline-regulated expression. The sequence of the native conserved inter-promoter sequence (blue) or its replacement derived from pSE100 (blue/black) is also shown (e).

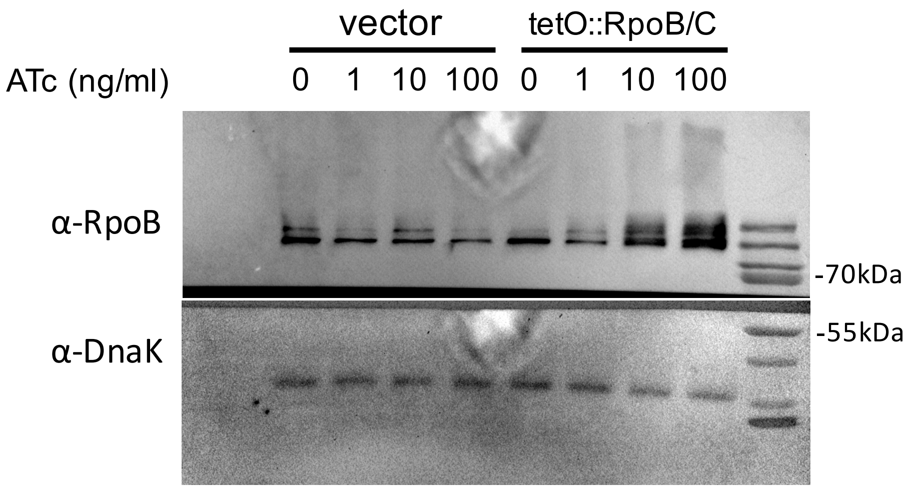

Supplementary Fig. 20. Full western blot of Fig. 3f

Supplementary Table 1. Quantitative-PCR primers used in this study.

Name	Sequence 5’-3’
Promoter-I-F	cgggttgaggacgccagc
Promoter-I-R	gaggtgggcagggtgcag
Promoter-II-F	ccacctcacctgtacctgac
Promoter-II-R	gcgactattcggatgcggca
rpoB-CDS-F (MSMEG_1367)	cgcttcggcgagatggaatg
rpoB-CDS-R (MSMEG_1367)	gcagctccttgagcaacacc
rpoC-CDS-F (MSMEG_1368)	agtcgctgtcggatctgctc
rpoC-CDS-R (MSMEG_1368)	atcgaccaggcgcttcatca
sigA-F (MSMEG_2758)	atccgtgcggtcgagaagtt
sigA-R (MSMEG_2758)	tggatacggccgagcttgtt
rpoB-CDS-F (Mb0686)	gttctctgaccctcgtttc
rpoB-CDS-R (Mb0686)	ggaagtcacccatgaacac
rpoC-CDS-F (Mb0687)	tgggatgtgctcgaagag
rpoC-CDS-R (Mb0687)	gcattgaacgcctcacac
sigA-F (MSMEG_2758)	gtacaagttctccacctacg
sigA-R (MSMEG_2758)	ggtgatgtccatctctttgg

Supplementary Table 2. Cloning primers used in this study.

Name	Sequence (5’-3’)
rpoB_FP_F	ggtctgctcggctcgacc

rpoB_mA_R	gccatgttattctcctcgcccttgctcgcgagatcctcgacggacgc
rpoB_mEm_R	cagctcctcgcccttgctcgcgagatcctcgacggacgc
rpoB_mA_F	agcaagggcgaggagaataacatggc
rpoB_mEm-F	agcaagggcgaggagctg
his_FP_R	cgaagttataggcctctcgaagtcagtgatggtgatggtgatgaca
FP_zeo_F	ccatcactgacttcgagaggcctataacttcgtata
FP_zeo_R	ccgagctcgaattaagtacttctagactcgagataacttcgtatagcat
ZeoR_rpoB_F	ctcgagtctagaagtacttaattcgagctcggagcctcgtccgaagaccg
rpoB_FP_R	gtcgtcgaccgaggtgat
rpoC_Nterm_R	cacgctgggcacggtcgcgc
SpeI_PrpoBC_F	tgcccttactagtatgcccagggtgatgccc
PrpoBC-F-1	atgcccagggtgatgccccggcccggcctgacgtgt
PrpoBC-F-2	atgcccagggtgatgccccgcgcgggcaaccgcgt
PrpoBC-F-3	atgcccagggtgatgcccgatgacggaggtgcgtgc
PrpoBC-F-4	atgcccagggtgatgcccgtccacacgacggtgccg
PrpoBC-F-5	atgcccagggtgatgcccggcacgcgggagtgggcg
PrpoBC-F-6	atgcccagggtgatgccctggcgggtggaacgtcac
PrpoBC-F-7	atgcccagggtgatgccccccaccaaacagcgtgt
PrpoBC-F-8	atgcccagggtgatgcccgttcgctcttgacggacg
PrpoBC-F-9	atgcccagggtgatgcccggacggccccggacgggc
PrpoBC-F-10	atgcccagggtgatgcccgacgggccaatctgttgg
PrpoBC-F-11	atgcccagggtgatgcccagcatgttcgaacgggtt
PrpoBC-F-12	atgcccagggtgatgcccggtgacccagctcatgcg
PrpoBC-F-13	atgcccagggtgatgcccgcccgaggggtttggttg
PrpoBC-F-14	atgcccagggtgatgcccaggaatgcgccgttctgc
PrpoBC-F-15	atgcccagggtgatgcccgttggacgttgcgctggc

PrpoBC-F-16	atgcccagggtgatgcccgcgctggctgcaccctgc
PrpoBC-F-17	atgcccagggtgatgcccctcacctgtacctgacac
PrpoBC-F-18	atgcccagggtgatgcccacaccgtggtcctagcct
PrpoBC-F-19	atgcccagggtgatgccctgagtagtttgctcagta
PrpoBC-R	gcccttgctgactgccaagatgcatccttccag
HindIII_FP_R	tagaaaccaagctttcagtgatggtgatggtgatg
PrpoBC-mEm-F	tcttggcagtcagcaagggcgaggag
PrpoBC-20-F	ggttgaggaatgcgccgttcgtagtttgctcagtacctgg
PrpoBC-20-R	ccaggtactgagcaaactacgaacggcgcattcctcaacc
Ptet-F	ttcattaatgcagctagaactagt
P21-tet-R	tcccagagcctatctatcactgatagggaggacaa
P22-tet-R	accaaaccccatttcgggcggcgaatctc
Promoter-II-21-F	gatagataggctctgggaagcatgttcgaacgggttga
Promoter-II-22-F	gcccgaaatgggggtttggttgaggaatgc
UVtet-mEm-R	taattagctaaagcttgatatctcagtgatggtgatggtgatg
PrpoBC_F	ggtggaacgtcacgcgag
PrpoBC_II_F	gaggacgccagccagcgc
Psmyc_F	aatattggatcgtcggcaccg
Psmyc_R	gcatgcggatcgtgctcatttc
1357-PromoterII-rpoB-F	gcggcgcttttttttttctcgactagtagcatgttcgaacgggtt
rpoB-rpoC-R	tcgtccgaagaccgcctcagtagtcaagtt
rpoB-rpoC-F	aacttgactactgaggcggtcttcggacga
1357-rpoC-R	atatttctagagtttaaacactagtttagcggtaatccgagtagccgta
rpoBC-KO-1	atcaacatcgcccgcaccgt
rpoBC-KO-2	gttataggcctctcgaaggcgtggaaatccggagaaaatc
rpoBC-KO-3	cttcgagaggcctataacttcgtataatgt

rpoBC-KO-4	tcgaattaagtacttctagactcgag
rpoBC-KO-5	gtctagaagtacttaattcgagtgtgccgaatgtgattacct
rpoBC-KO-6	gcacacctgcggttgcagggcagc
TB_BCP_F	taatactgtttaaacctctagacattctcacctgaggcaacg
TB_BCP_R	gtatatctccttcttaattaagatgcatccttccagcacttc
TB_BCP_P1_del_F	tcaacgcgaggacttgacggcagcatgcgccgcat
TB_BCP_P1_del_R	gtcaagtcctcgcgttgact
TBBCP_P2_del_F	atccggggatggttattgcgacgttgcgctggctact
TBBCP_P2_del_R	gcaataaccatccccggata

Supplementary Table 3. Plasmids used in this study.

<table>
<tr><th>Name</th><th>Selection Marker</th><th>Description</th><th>Reference</th></tr>
<tr><td>pML1342</td><td>Hyg</td><td>L5 site integration plasmid</td><td>[60]</td></tr>
<tr><td>pMV261-mApple</td><td>Hyg</td><td>Plasmid template of codon-optimized mApple.</td><td>Gift from Eric J. Rubin</td></tr>
<tr><td>pMV261-mEmerald</td><td>Hyg</td><td>Plasmid template of codon-optimized mEmerald.</td><td>Gift from Eric J. Rubin</td></tr>
<tr><td>pML1342-P1-mEmerald</td><td>Hyg</td><td rowspan="7">L5 integrating, rpoB-rpoC promoter variant driven mEmerald expression cassette.</td><td rowspan="7">This study</td></tr>
<tr><td>pML1342-P2-mEmerald</td><td>Hyg</td></tr>
<tr><td>pML1342-P3-mEmerald</td><td>Hyg</td></tr>
<tr><td>pML1342-P4-mEmerald</td><td>Hyg</td></tr>
<tr><td>pML1342-P5-mEmerald</td><td>Hyg</td></tr>
<tr><td>pML1342-P6-mEmerald</td><td>Hyg</td></tr>
<tr><td>pML1342-P7-mEmerald</td><td>Hyg</td></tr>
</table>

pML1342-P8-mEmerald	Hyg		
pML1342-P9-mEmerald	Hyg		
pML1342-P10-mEmerald	Hyg		
pML1342-P11-mEmerald	Hyg		
pML1342-P12-mEmerald	Hyg		
pML1342-P13-mEmerald	Hyg		
pML1342-P14-mEmerald	Hyg		
pML1342-P15-mEmerald	Hyg		
pML1342-P16-mEmerald	Hyg		
pML1342-P17-mEmerald	Hyg		
pML1342-P18-mEmerald	Hyg		
pML1342-P19-mEmerald	Hyg		
pML1342-P20-mEmerald	Hyg		
pUV15tetOR	Hyg	Tetracycline inducible multicopy plasmid	[61]
pUV15tetOR-P21-mEmerald	Hyg	Psmyc-tetO-Promoter-II-mEmerald with native inter-promoter sequence	This study
pUV15tetOR-P22-mEmerald	Hyg	Psmyc-tetO-Promoter-II-mEmerald with unrelated inter-promoter sequence	This study
pML1342-TB_BCP-mEmerald			
pML1342-TB_BCP-P1_del-mEmerald			
pML1342-TB_BCP-P2_del-mEmerald			
pML1357	Hyg	Giles site integration plasmid with P_{smyc} driven GFP expression	[60]

pML1357_Promoter II-rpoB-rpoC	Hyg	Giles site integration plasmid with rpoB-rpoC operon expressed from P_{rpoBC}-II.	This study
pMV261-EGFP*	Hyg	P_{hsp60} driven EGFP expression	This study
pNit(Kan)-RecET-SacB	Kan	IVN inducible RecET recombinase expressing cassette	[63]
pKM Zeo-lox	Zeo	Template for loxP_ZeoR_loxP sequence	[14]

Supplementary Table 4. *In vitro* transcription template sequences

**Full length rpoB-rpoC promoter (370bp)**
ggtggaacgtcacgcgagcgcaattcccccaccaaacagcgtgttcgctcttgacggacggccccggacgggccaatctgttgggcagcat<br>gttcgaacgggttgaggacgccagccagcgccgggtgacccagctcatgcgcccgaggggtttggttgaggaatgcgccgttctgcgctatc<br>gttggacgttgcgctggctgcaccctgcccacctcacctgtacctgacaccgtggtcctagcctgagtagtttgctcagtacctggtcctgtgtcg<br>ttgcgtccagggttctggacaggcccaagccagccgaaccgacgcagaatcgcgacagagatccggcgaatgccgcatccgaatagtcgc
**Promoter-I only (296bp)**
ggtggaacgtcacgcgagcgcaattcccccaccaaacagcgtgttcgctcttgacggacggccccggacgggccaatctgttgggcagcat<br>gttcgaacgggttgaggacgccagccagcgccgggtgacccagctcatgcgcccgaggggtttggttgaggaatgcgccgttcagtagtttg<br>ctcagtacctggtcctgtgtcgttgcgtccagggttctggacaggcccaagccagccgaaccgacgcagaatcgcgacagagatccggcgaa<br>tgccgcatccgaatagtcgc
**Promoter-II only (265bp)**
gaggacgccagccagcgccgggtgacccagctcatgcgcccgaggggtttggttgaggaatgcgccgttctgcgctatcgttggacgttgcg<br>ctggctgcaccctgcccacctcacctgtacctgacaccgtggtcctagcctgagtagtttgctcagtacctggtcctgtgtcgttgcgtccagggt<br>tctggacaggcccaagccagccgaaccgacgcagaatcgcgacagagatccggcgaatgccgcatccgaatagtcgc
**P_{smyc} (Control template, 309bp)**
aatattggatcgtcggcaccgtcacggccgtgggaggcggcacgatccgcgacgtgatgatcggccgcatccccacggtgctgcgcagtga<br>gctctacgccatcccggcgttgatctgtgcgttcgcacgcacaggcccggtgtgagaagggtctctgacgagcgggagaacccacccgggg

tgggcgagtttgtcctgcgtgtgctcggtcgagtaggctctgggagtacccgtgtgtacgaccagcacggcatacatcatttcgacgccgagag
attcgccgcccgaaatgagcacgatccgcatgc

Supplementary References

[63]van Kessel, J. C. & Hatfull, G. F. Mycobacterial recombineering. *Methods Mol. Biol.* **435**, 203–215 (2008).
