# Supplementary material for: Rifampicin can induce antibiotic tolerance in mycobacteria via paradoxical changes in rpoB transcription
Source: Nat Commun. 2018 Oct 11;9:4218. doi: 10.1038/s41467-018-06667-3 (PMC6181997; doi:10.1038/s41467-018-06667-3)
Supplement: Supplementary file 3 — Description of Additional Supplementary Files [file 41467_2018_6667_MOESM3_ESM.pdf]

Description of Additional Supplementary Files

File Name: Supplementary Movie 1
Description: Phenotypically resistant growers upregulate and accumulate RpoB. Time lapse video of the microcolony imaged in Fig. 3c.

File Name: Supplementary Movie 2
Description: Increased expression of rpoB and RpoB in response to rifampicin. Time lapse video of M. smegmatis expressing RpoB-mApple and PrpoB-rpoCmEmerald in a microfluidic chamber pre- and post-rifampicin infusion (representative images are shown in Fig. 4b).
